# Sex difference in mitral valve prolapse regurgitant volume is resolved by normalization of regurgitant volume to left ventricular end-diastolic volume

**DOI:** 10.1007/s10554-024-03197-6

**Published:** 2024-08-06

**Authors:** Chad M. House, Katie A. Moriarty, William B. Nelson

**Affiliations:** 1 Department of Cardiology, Regions Hospital, Chad House, 640 Jackson Street, Mail Stop 11102M, St. Paul, MN 55101 USA; 2 HealthPartners Medical Group, Minneapolis, MN USA; 3 University of Minnesota Medical School, Minneapolis, MN USA

**Keywords:** Sex differences, Mitral insufficiency, Mitral valve prolapse, Regurgitant fraction, Regurgitant volume

## Abstract

Women with primary mitral insufficiency have a smaller regurgitant volume at the same regurgitant fraction than men. We hypothesized that normalizing regurgitant volume with left ventricular end-diastolic volume or allometric scaling would eliminate the difference in regurgitant volume between women and men. The study cohort consisted of 101 patients with mitral valve prolapse undergoing cardiac MRI. Descriptive statistics and linear regression were performed to assess differences between sexes. Of the 101 patients, 46 (46%) were women. Women had a significantly smaller left and right ventricular end-diastolic volume, end-systolic volume, and stroke volume. While there was no difference in regurgitant fraction between women and men (34 ± 13% vs. 35 ± 14%; *p* = 0.71), women had a significantly smaller regurgitant volume (36 ± 18 ml vs. 49 ± 26 ml; *p* = 0.005). The slope-intercept relationship between regurgitant fraction and regurgitant volume revealed unique slopes and y-intercept values for men and women (*p*-value < 0.0001). Normalizing regurgitant volume to left ventricular end-diastolic volume (RVol/LVEDV), body surface area^1.5^ (RVol/BSA^1.5^) and height^2.7^ (RVol/height^2.7^) all had essentially identical slope-intercept relationships with regurgitant fraction for men and women, but RVol/LVEDV had the smallest effect size. In mitral insufficiency secondary to mitral valve prolapse women have a significantly smaller regurgitant volume than men despite no difference in regurgitant fraction. The significant difference in regurgitant volume between women and men is secondary to women having a smaller left ventricular end-diastolic volume.

## Introduction

We recently conducted a magnetic resonance imaging (MRI) study evaluating primary mitral insufficiency that found women have significantly smaller regurgitant volumes than men at the same regurgitant fraction [1]. These findings were subsequently confirmed by Altes et al. [2]. A regurgitant volume that is not concordant with regurgitant fraction by ACC and ESC guidelines may lead to mischaracterization of the severity of mitral insufficiency [3, 4]. The etiology of discordant values of regurgitant volume and regurgitant fraction between men and women has not been well characterized or discussed in guidelines.

Women with primary mitral insufficiency often have worse outcomes after mitral valve surgery than men, with increased mortality, morbidity, delayed referral for intervention, and less favorable left ventricular remodeling [5–11]. It has been hypothesized that the severity of women’s mitral insufficiency and left ventricular remodeling have been underestimated resulting in suboptimal timing for surgical referral.

Echocardiographic societal guidelines recognize that there are significant sex differences in left ventricular end-diastolic volume (LVEDV), left ventricular end-systolic volume (LVESV), and stroke volume (SV) [12]. These significant sex differences persist with indexing to body surface area (LVEDVi, LVESVi, SVi), however allometric scaling methods do correct differences between women and men [9–13]. Consistent with echocardiographic data, magnetic resonance imaging (MRI) normative data also reveal significant sex difference in left ventricular volumetric values [14]. In aortic stenosis, women have a significantly smaller mean gradient than men [15]. This is attributable to women having a smaller LVEDV and SV, as well as smaller body surface area.

We hypothesized that the sex difference in regurgitant volume in mitral valve prolapse (MVP) was secondary to differences in LVEDV and could be resolved by normalizing regurgitant volume to LVEDV. As an alternative, we hypothesized that allometric scaling would also explain sex differences in mitral regurgitant volume [13].

## Methods

The HealthPartners institutional review board approved, with a waiver of consent, a retrospective data review of cardiac MRI studies occurring between 1/1/2017 and 7/14/2023 with at least 10 ml of mitral insufficiency or a study indication of mitral insufficiency. Only patient’s ≥ 18 years of age were included. A database search (Synapse Cardiovascular, Fujifilm, Valhalla, NY, USA) identified an initial cohort of 1113 patients. The final study cohort included 101 patients with MVP.

Patients were excluded for the following reasons: atrial fibrillation during image acquisition; secondary mitral insufficiency (secondary to left ventricular dysfunction or an ischemic late gadolinium enhancement pattern); other significant valve disease (rheumatic mitral valve disease; > 15 ml of aortic insufficiency; aortic stenosis; > mild tricuspid insufficiency; valve replacement/repair); hypertrophic cardiomyopathy with systolic anterior motion of the mitral valve; congenital heart disease (intra-cardiac shunting; corrected or uncorrected congenital heart disease). Clinical and imaging data were abstracted through review of the patient’s electronic medical record (Epic, Verona, WI, USA).

## Magnetic resonance imaging

MRI was performed on a 1.5-T scanner (Aera or Sola, Siemens Medical Solutions, Erlangen, Germany). Image acquisition was performed during held expiration, with a body array coil and electrocardiographic gating. Interpretation was completed by one of four MRI trained cardiologists at our institution. Post-processing was performed with Medis Suite Cardiovascular Software (Vital Images, Eden Prairie, MN, USA) or Circle Cardiovascular Imaging (Calgary, AB, Canada) software beginning 6/1/2022. LVEDV, LVESV, right ventricular end-diastolic volume and right ventricular end-systolic volume were calculated from steady state free precision short-axis stack images, with a slice thickness of 8 mm and a 2 mm gap in accordance with image acquisition guidelines [16]. A 4-chamber view was used to correctly identify the mitral annulus (basal slice) during diastole and systole. The post-processing software then generated left ventricular end-diastolic and end-systolic epicardial and endocardial contours, and right ventricular endocardial contours (Circle CVI) on the short-axis stack images, which were manually adjusted, as needed. Papillary muscles, chordae tendineae and trabeculations were included within the blood pool. The difference between LVEDV and LVESV yielded left ventricular SV, which was then divided into LVEDV to determine left ventricular ejection fraction (these values were auto generated by the software once left ventricular contours were confirmed), and the difference between right ventricular end-diastolic volume and right ventricular end-systolic volume yielded right ventricular SV, which was then divided into right ventricular end-diastolic volume to determine right ventricular ejection fraction. Left atrial area was obtained by tracing the endocardial border in the 4-chamber view at end-systole. Phase contrast images were acquired perpendicular to the aorta just superior to the sinotubular junction, bisecting the pulmonary artery on a left ventricular outflow tract view during held end-expiration, with a slice thickness of 6 mm [16]. Velocity encoding was initially set at 150 cm/s and adjusted accordingly if aliasing present. Mitral regurgitant volume was calculated by subtracting the aortic SV, derived from the phase contrast images, from the volumetric left ventricular SV obtained from the short-axis stack data [17]. Regurgitant fraction was calculated by dividing the regurgitant volume into the left ventricular volumetric SV [17]. Left ventricular end-diastolic diameter (LVEDD) and left ventricular end-systolic diameter (LVESD) were measured in the left ventricular inflow/outflow view (akin to parasternal long-axis on echocardiography) as defined in the American Society of Echocardiography Guidelines, which has been validated for MRI [12, 18].

## Allometric methods

A previous publication identified BSA^1.5^ and height^2.7^ as allometric scaling indices validated for LVEDV [13]. Therefore, we elected to use these values for the allometric scaling of regurgitant volume.

## Statistics

Data are presented as mean ± standard deviation. A *p*-value < 0.05 was considered statistically significant. Comparisons were made using analysis of covariance, unpaired t-tests, Cohen’s *d,* and chi-square. Linear regression was performed to evaluate the strength and type of relationship between two independent variables or integrated variables. Analysis of covariance, with homogeneity of regression was used to compare the independent linear relationships for men and women.

## Results

Of the 101 patients, 46 (46%) were female. Women had significantly smaller myocardial mass, left ventricular volumes, right ventricular volumes and left atrial area (Table 1). There was no difference in regurgitant fraction between women and men (34 ± 13% vs. 35 ± 14%; *p*-value = 0.71), but women had significantly smaller regurgitant volumes (36 ± 18 ml vs. 49 ± 26 ml; *p*-value = 0.001).

**Table 1:** Demographic and magnetic resonance imaging data

	Male (55)	Female (46)	*p*-value
Age	66 ± 12	62 ± 15	0.13
BSA	2 ± 0.2	1.7 ± 0.2	< 0.0001
Heart Rate (bpm)	63 ± 10	68 ± 14	0.03
Myocardial mass (g)	145 ± 31	107 ± 22	< 0.0001
Myocardial mass index (g/m^2^)	71 ± 15	61 ± 11	0.0003
LVEDV (ml)	223 ± 48	169 ± 34	< 0.0001
LVESV (ml)	89 ± 27	67 ± 19	< 0.0001
LVEDVi (ml/m^2^)	110 ± 23	98 ± 19	0.005
LVESVi (ml/m^2^)	44 ± 13	39 ± 10	0.03
LVEF (%)	61 ± 6	61 ± 6	1.00
LV SV—volumetric (ml)	135 ± 28	102 ± 21	< 0.0001
LV SVI—volumetric (ml/m^2^)	66 ± 15	60 ± 12	0.03
Aortic SV (ml)	85 ± 20	66 ± 13	< 0.0001
Aortic SVI (ml/m^2^)	42 ± 10	39 ± 7	0.08
LVEDD (cm)	5.4 ± 0.8	5.1 ± 0.6	0.03
LVESD (cm)	3.4 ± 0.7	3.1 ± 0.6	0.02
RVEDV (ml)	191 ± 39	132 ± 25	< 0.0001
RVESV (ml)	91 ± 28	58 ± 15	< 0.0001
RVEF (%)	53 ± 6	56 ± 5	0.008
RV SV (ml)	100 ± 18	73 ± 13	< 0.0001
Left atrial area (cm^2^)*	33 ± 8	28 ± 7	0.0002
Mitral regurgitant volume (ml)	49 ± 26	36 ± 18	0.005
Mitral regurgitant fraction (%)	35 ± 14	34 ± 13	0.71
Regurgitant volume index (ml/m^2^)	24 ± 13	21 ± 11	0.21
Aortic regurgitant volume (ml)	4 ± 3	2 ± 2	0.0002

*BSA* body surface area, *LVEDV(i)* left ventricular end-diastolic volume (index), *LVESV(i)* left ventricular end-systolic volume (index), *LVEF* left ventricular ejection fraction, *LV SV(i)* left ventricular stroke volume (index), *SV(i)* stroke volume (index), *LVEDD* left ventricular end-diastolic diameter, *LVESD* left ventricular end-systolic diameter, *RVEDV* right ventricular end-diastolic volume, *RVESV* right ventricular end-systolic volume, *RVEF* right ventricular ejection fraction, *RV SV* right ventricular stroke volume

*Includes 47 men and 45 women

**Fig. 1:**
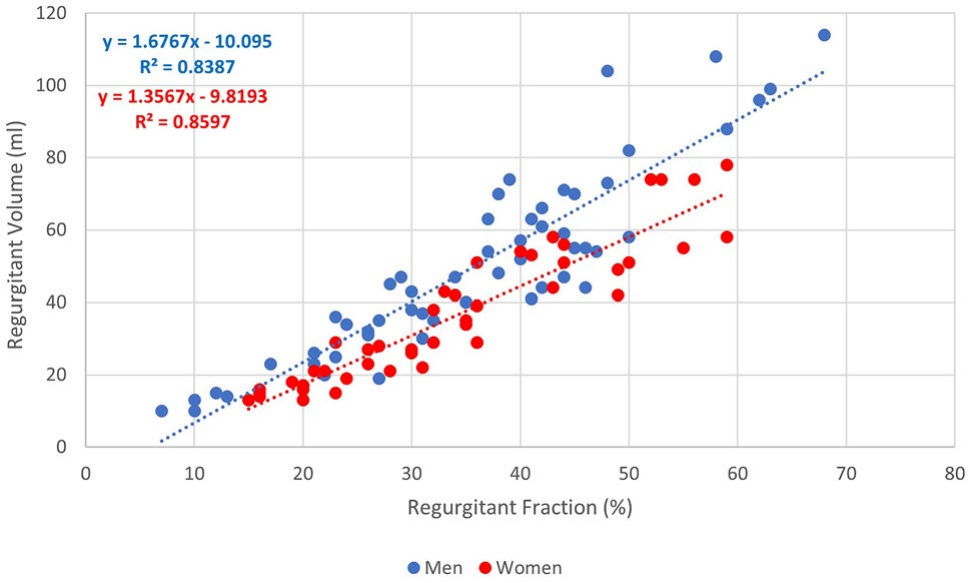
Sex differences in regurgitant volume at the same regurgitant fraction

Regurgitant fraction and regurgitant volume had a strong linear correlation for both women (r = 0.92) and men (r = 0.91). As shown in Fig. 1, linear regression identified unique slope-intercept relationships (ANCOVA < 0.0001 and homogeneity of regression = 0.02) between women (y = 1.35x − 9.8) and men (y = 1.67x − 10). The 95% confidence interval for slope was 1.19–1.52 in women and 1.47–1.87 in men. The 95% confidence interval for the y-intercept was − 15.8 to − 3.8 in women and − 17.8 to − 2.3 in men. A regurgitant fraction of 40% correlated with a regurgitant volume of 44 ml in women and 57 ml in men, while a regurgitant fraction of 50% correlated with a regurgitant volume of 58 ml in women and 74 ml in men.

### Left ventricular remodeling

The correlation of regurgitant volume with LVEDV was 0.60 in women and 0.61 in men, while the correlation with LVEDVi was 0.62 in women and 0.65 in men. The correlation between regurgitant volume and LVEDD was 0.37 in women and 0.21 in men. Regurgitant volume and LVESV had a correlation of 0.22 in women and 0.32 in men. Regurgitant volume and LVESD had a correlation of 0.13 in both women and men.

### Indexing regurgitant volume

**Fig. 2:**
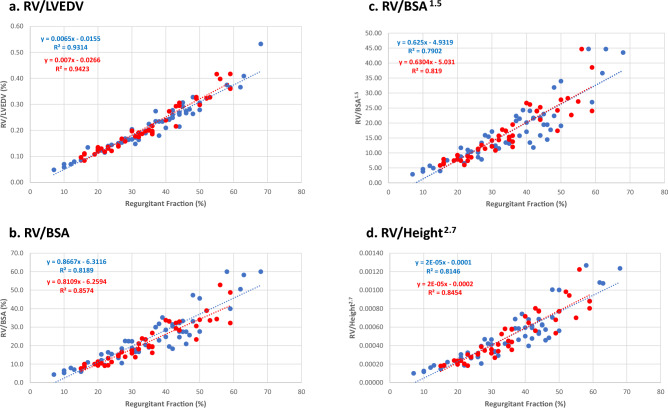
a–d Indexing regurgitant volume to left ventricular end-diastolic volume and allometric indices

We compared regurgitant volume indexed to body surface area (RVol/BSA), BSA^1.5^ (RVol/BSA^1.5^), height^2.7^ (RVol/height^2.7^), and LVEDV (RVol/LVEDV) with regurgitant fraction to determine which best resolved the difference in regurgitant volume between women and men. The ratio RVol/LVEDV had similar slope-intercept relationships with regurgitant fraction for men and women (Fig. 2a), with statistically indifferent slopes (ANCOVA = 0.26) and y-intercepts (homogeneity of regression = 0.25). The central figure highlights the different regurgitant volumes and LVEDV for women and men at the same regurgitant fraction and then shows the resolution of differences in regurgitant volume once normalized for LVEDV. The slopes and y-intercepts for regurgitant fraction and RVol/BSA (Fig. 2b), RVol/BSA^1.5^ (Fig. 2c) and RVol/height^2.7^ (Fig. 2d) were also statistically indifferent for men and women (p = 0.07 and 0.48; 0.92 and 0.92; 1 and 0.60). As shown in Table 2, RVol/LVEDV had the smallest effect size between women and men.

**Table 2:** Effect size between indexed regurgitant volume indices

	Men	Women	*p*-value	Cohen's *d*
RV/BSA	24 ± 13	21 ± 11	0.21	0.249
RV/BSA^1.5^	17 ± 10	16 ± 9	0.60	0.105
RV/Height^2.7^	0.00050 ± 0.00028	0.00047 ± 0.00026	0.58	0.111
RV/LVEDV	0.21 ± 0.09	0.21 ± 0.09	1	0

### Right ventricle

**Fig. 3:**
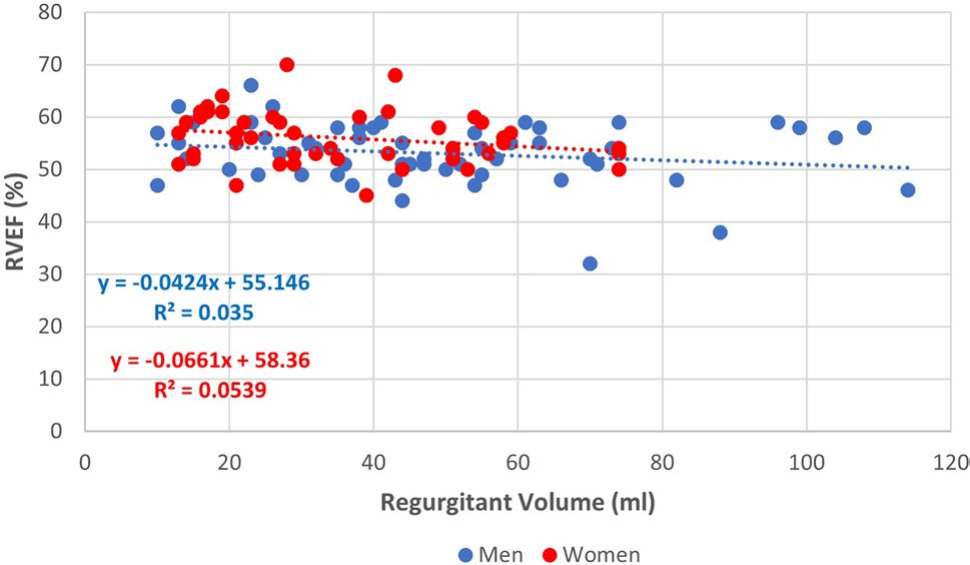
Relationship between regurgitant volume and right ventricular ejection fraction

There was no statistically significant relationship between regurgitant volume and right ventricular end-diastolic volume in either women (r = 0.15) or men (r = 0.07). Regurgitant volume, regurgitant fraction, and RVol/LVEDV all had a weak, inverse linear relationship with right ventricular ejection fraction in both women (r = − 0.23, − 0.21, − 0.18) and men (r = − 0.18, − 0.25, − 0.13), with the relationship between regurgitant volume and right ventricular ejection fraction shown in Fig. 3.

### Left atrium

**Fig. 4:**
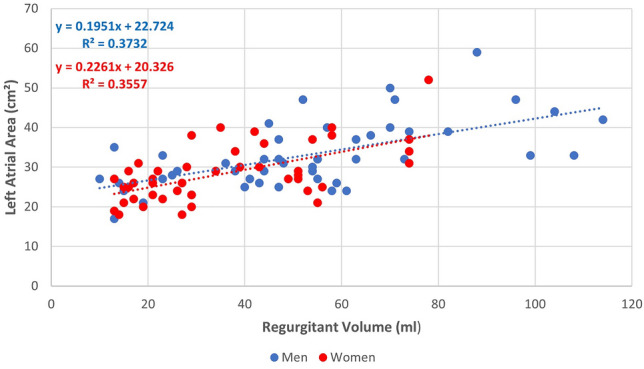
Relationship between regurgitant volume and left atrial area

A linear relationship was observed between regurgitant volume and left atrial area in both women (r = 0.59) and men (r = 0.61), as shown in Fig. 4. The correlation between regurgitant fraction and left atrial area was 0.46 in men and 0.52 in women, while the correlation between RV/LVEDV and left atrial area was 0.44 in men and 0.47 in women.

### Forward stroke volume

**Fig. 5:**
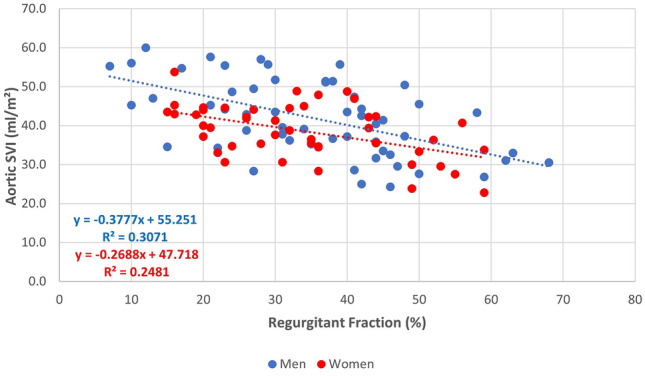
Relationship between regurgitant fraction and aortic stroke volume index

An inverse linear relationship was observed between regurgitant fraction and aortic SV in both women (r = -0.44) and men (r = -0.56), and aortic SVi with an r = 0.49 in women and r = 0.55 in men (Fig. 5). As shown in Table 3, both men and women with regurgitant fractions ≥ 50% had an average aortic SVi ≤ 35 ml/m^2^.

**Table 3:** Aortic stroke volume relative to mitral regurgitant fraction in men and women

Regurgitant fraction (%)	Men		Women	
	Aortic SV	Aortic SVI	Aortic SV	Aortic SVI
< 20	103 ± 17	50 ± 8	77 ± 5	46 ± 4
20–29	94 ± 19	47 ± 9	68 ± 13	39 ± 5
30–39	91 ± 15	44 ± 7	67 ± 13	39 ± 6
40–49	77 ± 15	37 ± 7	64 ± 13	39 ± 8
> 50	64 ± 10	34 ± 7	54 ± 10	32 ± 6
*p*-value	< 0.001	< 0.001	0.03	0.007

There were 14 patients that had a regurgitant fraction ≥ 50% (7 women and 7 men). Of the men, 6 (86%) had left ventricular enlargement (LVEDVi ≥ 108 ml/m^2^), while 5 (71%) of the women had left ventricular enlargement (LVEDVi ≥ 96 ml/m^2^) [14]. All 3 patients (1 man and 2 women) without left ventricular enlargement had a very low aortic SVi (27.5 and 22.8 ml/m^2^ in the women and 27.6 ml/m^2^ in the man). There were 4 (57%) men and 3 (43%) women that had both left ventricular enlargement and a reduced SVi. All 14 patients with a regurgitant fraction ≥ 50% had either left ventricular enlargement or a decreased aortic SVi, and 50% had both.

There were 25 patients that had regurgitant fraction ≥ 40% but less than 50% (17 men and 8 women). Of the men, 9 (53%) had left ventricular enlargement, while 6 (75%) of the women had left ventricular enlargement. There were 7 (41%) men that had an aortic SVi ≤ 35 ml/m^2^, and none had left ventricular enlargement. There were 2 (25%) women that had an aortic SVi ≤ 35 ml/m^2^, and 1 also had left ventricular enlargement. Of the 25 patients with a regurgitant fraction ≥ 40% and less than 50%, 23 (92%) had either left ventricular enlargement or a decreased aortic SVi.

## Discussion

Key findings in chronic primary mitral insufficiency secondary to prolapse are:

1. The sex difference in regurgitant volume secondary to MVP can be resolved by normalizing RVol/LVEDV.
2. Regurgitant fraction, RVol/LVEDV, or both are sex-independent tools for determining severity of primary mitral insufficiency.
3. Allometric scaling of regurgitant volume with height and body surface area also resolves the sex difference, but not as closely as RVol/LVEDV.
4. As regurgitant volume increases, LVEDV increases, but with women having smaller absolute LVEDV values.
5. As the regurgitant volume increases, there is a corresponding decrease in aortic SV for both sexes, but with women having smaller absolute SV values.

## Left ventricular remodeling

Both men and women progressively enlarge their left ventricle as the degree of mitral insufficiency increases, but women’s absolute LVEDV remains consistently smaller than men’s [1, 2]. This causes a smaller SV and regurgitant volume for women. The ratio RVol/LVEDV has essentially an identical slope-intercept relationship with regurgitant fraction for men and women. This identifies that the difference in regurgitant volume between women and men is attributable to women having a smaller LVEDV than men. Allometric scaling of height and body surface area also normalizes sex differences in regurgitant volume, but not as closely as RVol/LVEDV.

**Figure:**
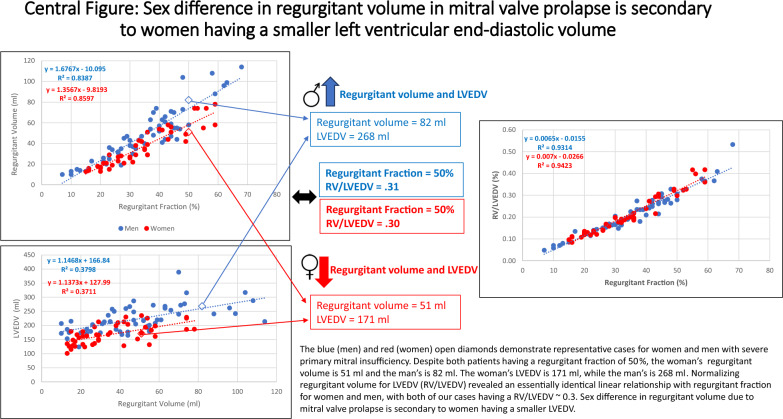
Central figure

As primary mitral insufficiency is a preload problem, progressive left ventricular enlargement is a cardinal feature. This is reflected in our data, as the left ventricular parameter that has the strongest correlation with regurgitant volume is LVEDV. A recent publication found that as primary mitral insufficiency severity increased MRI derived mitral regurgitant volume best predicted left ventricular enlargement, but with different optimal cut-points for men and women [2]. Left ventricular reverse remodeling after mitral intervention has an inverse, linear relationship with MRI derived regurgitant volume [20].

Guidelines endorse LVESD as a surgical threshold [2, 3]. Multiple MRI publications have identified a weak correlation between LVESD and regurgitant volume, our data are consistent with this [1, 2, 19, 20]. Reaching or exceeding a LVESD of 4 cm has good specificity for predicting post-operative left ventricular dysfunction in primary mitral insufficiency, but sensitivity is suboptimal at 60% [21]. In our study, only 1 of the 15 women with a regurgitant fraction ≥ 40% had a LVESD ≥ 4 cm. These data, along with previous studies, imply that LVESD will often underestimate left ventricular remodeling in women despite significant primary mitral insufficiency [2, 20].

These data suggest that LVEDV is the most sensitive left ventricular parameter to identify significant primary mitral insufficiency. The goal of mitral intervention is to reduce the morbidity and mortality associated with chronic primary mitral insufficiency, but the optimal time to intervene is not clear. A LVESD ≥ 4 is almost certainly not optimal for most women, and some men. Sex-specific left ventricular enlargement by LVEDV or LVEDVi provides a more sensitive assessment of the volumetric overload of chronic primary mitral insufficiency but has not been validated clinically.

## Aortic stroke volume

We identified an inverse, linear relationship between regurgitant volume and aortic SV. The 7 women with a regurgitant fraction ≥ 50% had an average aortic SV of 54.3 ml. A recent study identified a strong linear relationship between LVEDV and VO_2_ max in 185 women [22]. In this study, LVEDV quartiles were strongly associated with cardiac reserve, and the lowest LVEDV quartile had a SV of ~ 70 ml and a maximal cardiac output of approximately 12 L/min. Based on these data, we hypothesize that our 7 patients with severe primary mitral insufficiency and an average aortic SV of 54 ml probably have a very low maximal cardiac output and VO_2_ max. Thus, it is plausible that a reduced aortic SV in primary mitral insufficiency is a strong contributor to the symptom of dyspnea on exertion, with the mechanism being an early arrival at their anaerobic threshold.

A reduced aortic SV may provide an objective variable that demonstrates the hemodynamic impact of severe primary mitral insufficiency. Aortic SV appears to be a useful parameter in the assessment of primary mitral insufficiency severity with the potential to contribute to intervention timing in mitral valve prolapse patients.

## Grading primary mitral insufficiency

Women have a smaller LVEDV than men and generate a smaller SV [12, 14]. A reduced flow rate has been recognized as the reason why women have a smaller mean gradient than men in aortic stenosis [15]. This same concept affects the regurgitant volume in mitral insufficiency, with our data showing that women have a smaller volumetric SV, aortic SV and mitral regurgitant volume. Despite the significant disparity in regurgitant volume between women and men, there is no difference in regurgitant fraction, which is attributed to the smaller LVEDV, as demonstrated in the central figure.

Cardiac MRI derived regurgitant fraction has been found to be reliably associated with NYHA functional class III/IV, significant left atrial enlargement, and pulmonary hypertension regardless of sex [2]. An MRI derived regurgitant fraction ≥ 40% has been identified as a prognostic threshold in primary mitral insufficiency, but, to date, there are no MRI guidelines for the grading of mitral insufficiency severity [23].

Like regurgitant fraction, the ratio RVol/LVEDV also is a sex independent measurement of mitral insufficiency severity, with a ratio of 0.26 and 0.32 correlating with a regurgitant fraction of 40% and 50% in our data. This is consistent with a previous publication that identified a RVol/LVEDV of 0.3 corresponding with a regurgitant fraction of 50% in primary mitral insufficiency by echocardiographic data [24]. Regurgitant fraction and RVol/LVEDV are interdependent, with the difference being the denominator for RVol/LVEDV is LVEDV versus LVEDV–LVESV for regurgitant fraction. Either measurement could be used to determine severity of primary mitral insufficiency, however in current clinical practice regurgitant volume is commonly used, which can mischaracterize the severity of mitral insufficiency, particularly for women. Use of both regurgitant fraction and RVol/LVEDV to determine severity of mitral insufficiency in mitral valve prolapse provides reassurance when regurgitant volume and regurgitant fraction are disparate.

## Limitations

These data are retrospective, which introduces the potential for selection bias. It is reassuring that other publications have also found similar data regarding sex differences in primary mitral insufficiency [2, 25]. There are no clinical data included with this study to evaluate for any outcome related differences according to sex.

## Conclusion

Regurgitant fraction and RVol/LVEDV provide sex independent assessment of mitral insufficiency severity, while regurgitant volume requires adjustment for sex. The sex difference in regurgitant volume is secondary to women having a smaller left ventricular volume than men. An increased LVEDV and reduced aortic stroke volume reflect the sequala of chronic significant primary mitral insufficiency, but with sex-specific values. Regurgitant fraction, RVol/LVEDV, LVEDV and aortic stroke volume may provide optimal MRI parameters for objectively determining mitral insufficiency severity and potentially intervention timing.

## Data Availability

Data will be provided upon reasonable request to the corresponding author.

## Abbreviations

BSA: Body surface area
LVEDD: Left ventricular end-diastolic diameter
LVEDV(i): Left ventricular end-diastolic volume (index)
LVESD: Left ventricular end-systolic diameter
LVESV(i): Left ventricular end-systolic volume (index)
MRI: Magnetic resonance imaging
MVP: Mitral valve prolapse
RVol/BSA(^1.5^): Regurgitant volume/body surface area (^1.5^)
RVol/height(^2.7^): Regurgitant volume/height(^2.7^)
RVol/LVEDV: Regurgitant volume/left ventricular end-diastolic volume
SV(i): Stroke volume (index)
